# Network Pharmacology Reveals the Potential Mechanism of Ketamine in Improving Cognitive Impairment in Patients With Depression

**DOI:** 10.31083/AP56752

**Published:** 2026-08-06

**Authors:** Lei Yang, Qiuyu Zhang, Ying Zhang, Kaifang Yao, Ximing Chen, Hongjun Tian, Chuanjun Zhuo

**Affiliations:** ^1^Computational Biology and Animal Imaging Center (CBAC), Tianjin Anding Hospital, Nankai University Affiliated Tianjin Anding Hospital, Tianjin Medical University Affiliated Tianjin Anding Hospital, Tianjin Medical University Affiliated Tianjin Mental Health Center, 300222 Tianjin, China; ^2^Laboratory of Psychiatric-Neuroimaging-Genetic and Co-morbidity (PNGC_Lab), Tianjin Anding Hospital, Tianjin Mental Health Center of Tianjin Medical University, 300222 Tianjin, China; ^3^Department of Psychiatry, Tianjin Fourth Center Hospital, Nankai University Affiliated Tianjin Fourth Center Hospital, 300140 Tianjin, China

**Keywords:** ketamine, depression, cognitive impairment, network pharmacology, molecular docking

## Abstract

**Background:**

Ketamine may have antidepressant and anti-suicidal effects. However, the mechanism underlying ketamine-mediated improvement in cognitive impairment in patients with depression remains unclear. To improve patient cognition in depression using molecular docking and network pharmacology, we examined ketamine's key targets and identified its molecular mechanisms.

**Methods:**

To gain target information about cognitive impairment (1983) and depression (1874), we used three databases, including Online Mendelian Inheritance in Man, GeneCards, and DisGENET. Information on ketamine targets (63) was retrieved from public databases. To locate signaling pathways and core targets, we conducted bioinformatics analysis, including an enrichment analysis and protein–protein interaction (PPI) network analysis. To assess the interaction between core targets and ketamine, we carried out molecular docking.

**Results:**

We identified 20 ketamine target proteins of ketamine related to depression and cognitive impairment. Enrichment analyses revealed that ketamine influenced depression and cognitive function through multiple pathways, targets, and overall synergy. The important signaling pathways identified were “amphetamine addiction” and “dopaminergic synapse”. Five core genes (monoamine oxidase (MAO)-A, *MAO-B*, *glycogen synthase kinase-3β*, *sirtuin-1*, *epidermal growth factor receptor*) were identified through PPI-network analyses. Our molecular docking results showed that binding was strong between these core genes and ketamine.

**Conclusions:**

The antidepressant and cognitive-enhancing effects of ketamine are primarily mediated through targets associated with inflammation, neural signaling, tumors, and neurodegeneration, as well as pathways such as amphetamine addiction, dopaminergic synapse, and the cyclic adenosine monophosphate (cAMP) signaling pathway. The findings provide theoretical support and guidance for optimizing clinical application strategies of ketamine and for designing new drugs.

## Main Points

1. Multiple targets and signaling pathways are involved in cognitive impairment in depression.

2. The amphetamine addiction, dopaminergic synapse, cyclic adenosine monophosphate (cAMP), Ras-association proximate 1 (Rap1), and Ras signaling pathways may be crucial factors in the development of cognitive impairment in depression.

3. Ketamine may alleviate cognitive impairment in patients with depression by targeting multiple pathways, including amphetamine addiction, dopaminergic synapse, cAMP signaling, Rap1 signaling, and Ras signaling pathways.

## 1. Introduction

Cognitive impairment (CI) in patients with depression is mainly involved in executive function, attention, memory, and speed of information processing. Any stage of major depressive disorder (MDD) can feature symptoms of CI. CI is a critical issue in depression due to its high prevalence and substantial impact on patients’ functional outcomes and prognosis. In the acute stage, the prevalence of CI is between 76.9% and 94.0%, and in the remission stage, it is between 32.4% and 44.0%, according to past research [1,2]. CI often leads to poor prognosis when observed in the presence of depression [3,4]. Despite advances in understanding depression, effective treatments specifically targeting CI remain limited. Therefore, it is crucial to explore novel therapeutic strategies to improve CI in depression. Because of its analgesic and anesthetic properties, ketamine has been in use since the 1970s [5]. Mounting pre-clinical studies converged to report that ketamine can rapidly improve the depressive symptoms in animal models [6].

CI in depression involves complex neural circuits, including both top-down control by the cerebral cortex and bottom-up influences from regions such as the hippocampus and amygdala [7,8]. In depression, changes in the release of monoamines may lead to decreased cognitive control from the top down and increased signaling from the bottom up, which may further trigger negative patterns of behavior and cognition [9,10].

Ketamine has received a lot of attention because of its robust and rapid effects as an antidepressant. According to research, one dose of ketamine, which is a noncompetitive N-methyl-D-aspartate (NMDA) receptor antagonist, can alleviate symptoms of depression within hours in patients who have MDD. The effects of ketamine can also last for several days. Selective serotonin reuptake inhibitors (SSRIs), which are traditional antidepressants, do not operate like ketamine, which has a distinct mechanism and a rapid onset of action. Therefore, ketamine is particularly effective for depression that has been resistant to other treatments [10,11]. In addition to its antidepressant properties, ketamine also has been found to improve CI [12,13,14]. Previous studies have shown that ketamine may enhance synaptic plasticity and promote neurogenesis, contributing to improvements in cognitive domains such as executive control, memory, and attention [12]. The effects of ketamine on CI, however, have not been explored in depth. Its long-term safety and potential side effects have not been studied extensively. Therefore, in this study, we examined the mechanisms by which ketamine may improve CI in patients who have depression by applying a network pharmacology approach. The results of this study thus provide novel insight into potential clinical applications of ketamine.

Using computational prediction, network pharmacology is able to reveal the biological mechanisms that underlie complex diseases as well as the molecular impact of pharmaceuticals [15]. In the past, network pharmacology was used primarily to forecast the molecular mechanisms of drugs [16,17,18].

**Fig. 1.**
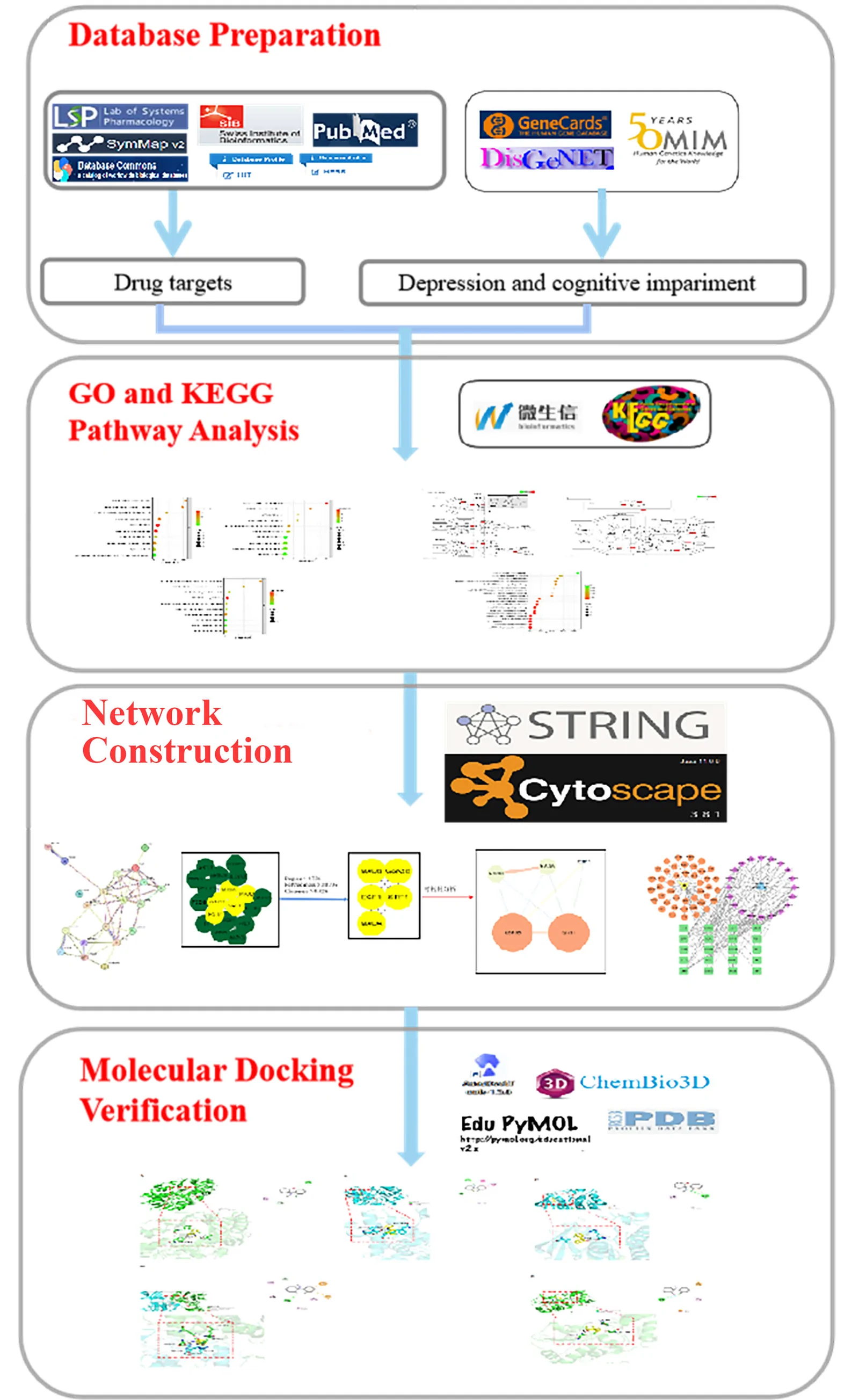
**Workflow of the present study**. Network pharmacology was employed to investigate the antidepressant and cognition-improvement functions of ketamine, and comprised four parts: database preparation; enrichment analyses of the functional and signaling pathways of genes using the Gene Ontology (GO) and Kyoto Encyclopedia of Genes and Genomes (KEGG) databases; network construction; and verification by molecular docking.

We revealed the molecular pathways and main targets through which ketamine can enhance CI in patients with depression. This present study contributes to a greater understanding of the therapeutic potential of ketamine. Fig. 1 illustrates the study design.

## 2. Methods

### 2.1 Data Collection

#### 2.1.1 Screening of the Potential Targets of Ketamine

Drug targets for ketamine compounds were directly screened using the Traditional Chinese Medicine Systems Pharmacology database (TCMSP: https://tcmsp-e.com/), Symptom Mapping database (SymMap: http://www.symmap.org/) [19], a high-throughput experiment- and reference-guided database of traditional Chinese medicine (HERB: http://herb.ac.cn/Search/) [20], and Herbal Ingredients’ Targets Platform (HIT: http://www.badd-cao.net:2345/) [21]. To search for the Simplified Molecular Input Line Entry System number of ketamine, we used the PubChem database (https://pubchem.ncbi.nlm.nih.gov/) [22]. We entered this number into the SwissTargetPrediction database (http://SwissTargetprediction.ch/) [23] so that we could predict targets and remove duplicates of ketamine. We collated all of the drug targets of ketamine that we obtained from these databases. After we removed the duplicate targets, we were able to identify ketamine’s possible drug action targets.

#### 2.1.2 Acquisition of the Targets for Depression and CI

We used three databases to collect data for the target diseases (i.e., depression and CI), namely DisGENET (https://disgenet.com/), Online Mendelian Inheritance in Man (OMIM; http://omim.org) [24], and GeneCards (https://www.genecards.org/) [25]. “Cognitive impairment” and “depression” were our keywords. Each keyword was queried separately to ensure retrieval of disease-related targets. The results of database searches were combined to obtain all of the targets for depression and CI. A gene-target library of depression and CI was established by eliminating targets that were duplicated. To refine the dataset, we manually validated the relevance of each target to depression and CI based on database annotations and a literature review.

#### 2.1.3 Intersection of Drug Ingredients and Disease Targets

To make a Venn diagram highlighting the potential targets of ketamine and their relationship to CI and depression, we used Venny 2.1.0 (https://bioinfogp.cnb.csic.es/tools/venny/index.html) [26]. We analyzed the points at which these areas intersected. We noted that ketamine had 20 potential targets that contributed to its role in improving CI and depression.

### 2.2 Construction of a Protein–Protein Interaction Network and Topological Analysis

We used the Search Tool for the Retrieval of Interacting Genes/Proteins (STRING) database (https://cn.string-db.org/) to identify the gene targets of CI, depression, and ketamine for interactions with protein [27]. We selected *Homo sapiens* (Human) as the species for analysis (confidence score ≥0.4). Cytoscape 3.7.1 (https://cytoscape.org/) identified degree centrality, proximity centrality, and intermediate centrality as the key topological parameters [28]. To develop the screening core targets and potential core-target network, we used these three parameters.

### 2.3 Kyoto Encyclopedia of Genes and Genomes and Gene Ontology Analyses

We used the Database for Annotation, Visualization and Integrated Discovery (https://ngdc.cncb.ac.cn/) and entered 20 target proteins of ketamine [29]. Considering the context of antidepression and cognition improvements, we analyzed the enrichment of the signaling and functional pathways of these genes. We used the Kyoto Encyclopedia of Genes and Genomes (KEGG) database (https://www.genome.jp/) and the Gene Ontology (GO) database (https://geneontology.org/). We screened the cellular component (CC), molecular function (MF), and biological process (BP) for enrichment results. The false discovery rate was <0.05 and *p* < 0.05. According to the number of enrichment items, we visualized the top-10 functions and the top-20 signaling pathways.

### 2.4 Construction of the Network

To empirically evaluate the complex mechanism of ketamine in treating depression and improving cognition, we imported the screening results of ketamine and the targets of depression and CI into Cytoscape 3.7.1 (https://cytoscape.org/). We developed a comprehensive network diagram, which we called the “drug–target–disease pathway”, using this software.

### 2.5 Molecular Docking Verification

We used the PubChem database (https://pubchem.ncbi.nlm.nih.gov/) to download the ketamine compound [22]. We used the Protein Database (PDB: https://www.rcsb.org/) to search for and obtain five proteins [30]. We docked the ketamine compound with these proteins, which we then defined as the “core targets”. We used AutoDock Vina 4.2 (https://vina.scripps.edu/) and AutoDockTools 1.5.6 (https://autodock.scripps.edu/) to carry out the following five steps involved in molecular docking [31]:

1. We downloaded the file for the three-dimensional structure of ketamine from the PubChem database to calculate the hydrogenation of the three-dimensional structure. We saved the results as a pdbqt file before we used AutoDockTools 1.5.6.

2. We obtained the crystal structures of proteins from the PDB protein library. After we imported the results into PyMOL 2.4.1 (https://pymol.org/2/), we removed the ligands. We saved the results for the structures in pdb format. We added hydrogen atoms to process the protein structures. Then, we calculated the charges and assigned the types of atoms. We save these structures for molecular docking in pdbqt format.

3. We used AutoDockTools 1.5.6 to construct the docking grid box for each target protein’s active site. We saved the grid box in pdbqt format.

4. To complete the molecular docking of ketamine and the target proteins, we used AutoDock Vina 4.2. We assessed the docking scores and determined the binding activity. If the docking score was less than –7, we determined it had “good” binding activity. If the docking score was less than –9, we determined it had a “strong” binding ability [31].

5. We used PyMOL 2.4.1 and Discovery Studio 2021 (https://discover.3ds.com/discovery-studio-visualizer-download) to visualize and assess the binding and interaction mode of the active compounds.

## 3. Results

### 3.1 Potential Target Genes of Ketamine

Target modules were retrieved from the TCMSP, SymMap, HERB, HIT, and SwissTargetPrediction databases, identifying 63 potential targets of ketamine, including the glutamate NMDA receptor, poly [ADP-ribose] polymerase-1, melatonin receptor 1A, glycogen synthase kinase-3 beta, metabotropic glutamate receptor 4, vascular endothelial growth factor receptor 3, insulin-like growth factor I receptor, and EGFR erbB1, among others, as well as their corresponding gene symbols, including glutamate receptor ionotropic N-methyl-D-aspartate-1 (*GRIN1*), poly ADP-ribose polymerase 1 (*PARP1*), melatonin receptor 1A (*MTNR1A*), glycogen synthase kinase 3beta (*GSK-3β*), glutamate metabotropic receptor 4 (*GRM4*), Fms-related tyrosine kinase-4 (*FLT4*), insulin-like growth factor-1 receptor (*IGF1R*), and epidermal growth factor receptor (*EGFR*), among others.

### 3.2 Target Genes for Depression and CI

A total of 1874 targets associated with depression and 1983 targets linked to CI were obtained by integrating the targets related to depression and CI screened from the databases of GeneCards, OMIM, and DisGeNET. Empty and duplicate targets were removed. We used the Universal Protein (UniProt: https://www.uniprot.org/) database to convert the protein target names to the corresponding gene symbol.

### 3.3 Common Targets of Ketamine, Depression, and CI

**Fig. 2.**
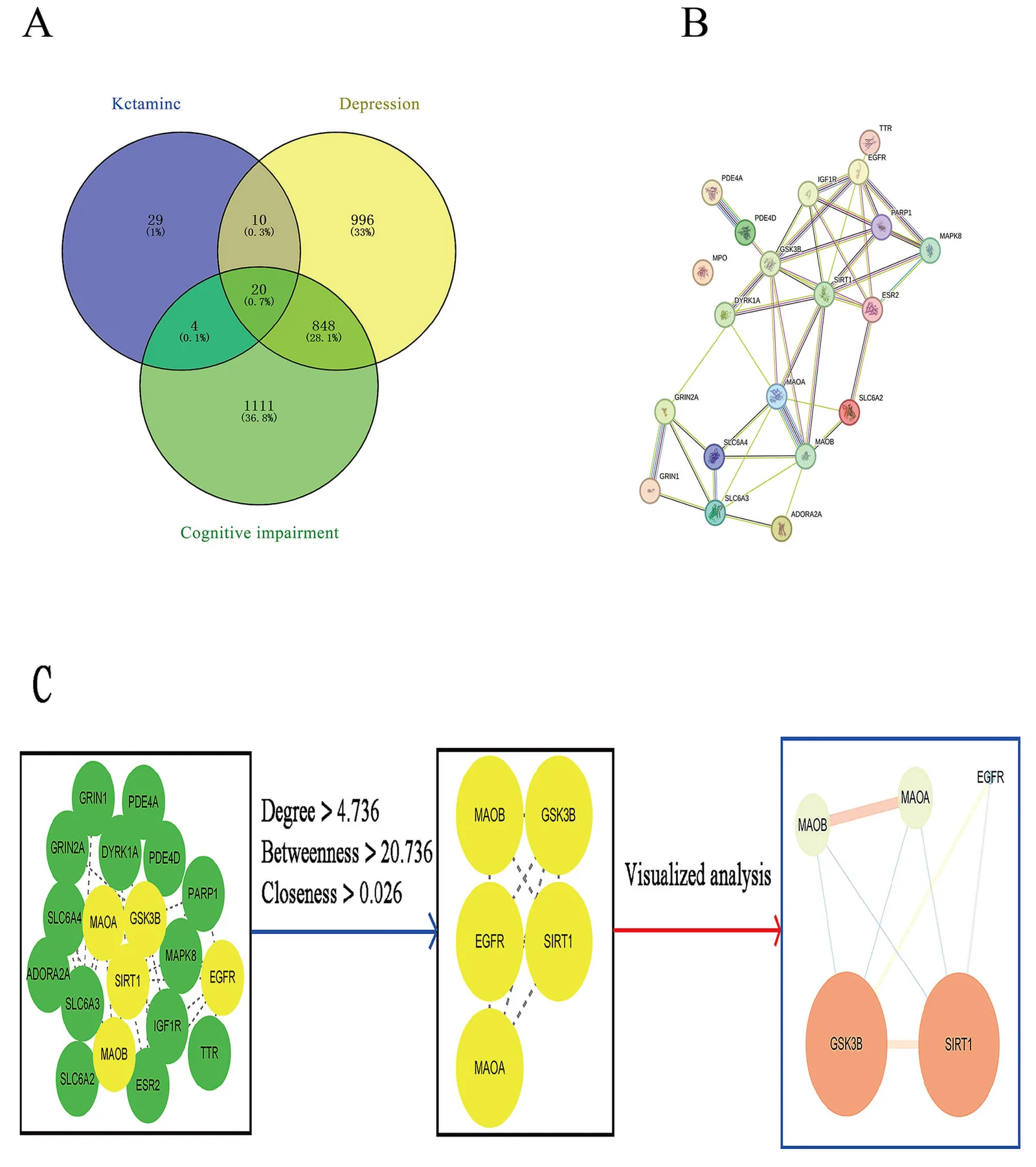
**Ketamine - common targets for depression and cognitive impairment, and screening for core targets in the PPI network by means of topological analysis**. (A) Venn diagram showing the targets of ketamine, as well as the targets of depression and cognitive impairment. (B) Potential targets were obtained via protein-protein interaction (PPI) analysis. (C) Topological screening process for the PPI network. The five core targets were obtained by screening 20 common targets through degree centrality (DC), intermediate centrality (BC), and proximity centrality (PC).

We overlapped the targets associated with depressive disorders (DDs) and CI, as shown in Fig. 2A. Then, we used the Venny platform to identify 20 targets common to ketamine in DD and CI treatment. The 20 shared targets were as follows: *GRIN1*, *GRIN2A*, *pARP1*, *GSK-3β*, *PDE4A*, *pDE4D*, *IGF1R*, *EGFR*, *MAOB*, *MPO*, *MAOA*, *SIRT1*, *ADORA2A*, *DYRK1A*, *MAPK8*, *SLC6A2*, *SLC6A4*, *SLC6A3*, *ESR2*, and *TTR*.

### 3.4 Protein–Protein Interaction Network of Common Targets

We entered 20 genes into the STRING database so that we could assess the interactions between ketamine and depression and between ketamine and CI. We selected *Homo sapiens* (Human) as the species for analysis (confidence score ≥0.4). We ended up with a protein–protein interaction (PPI) network that had 19 nodes with 45 edges. Each of the nodes represented a protein, and each edge represented an interaction with the nodes, as shown in Fig. 2B. To analyze the core network and targets further, we used the “centiscape2.2” plugin (http://apps.cytoscape.org/apps/centiscape) of Cytoscape 3.7.1. We calculated three parameters (degree, closeness, betweenness) for each node in the network. We selected only the nodes that met the criteria of degree >4.736, closeness >0.026, and betweenness >20.736. A new core PPI network as well as core targets were extracted, including the following five nodes: monoamine oxidase (MAO)-A, MAO-B, glycogen synthase kinase (GSK)-3β, epidermal growth factor receptor (EGFR), and sirtuin-1 (SIRT1), as shown in Fig. 2C.

### 3.5 Functional Enrichment of Genes Analysis

**Fig. 3.**
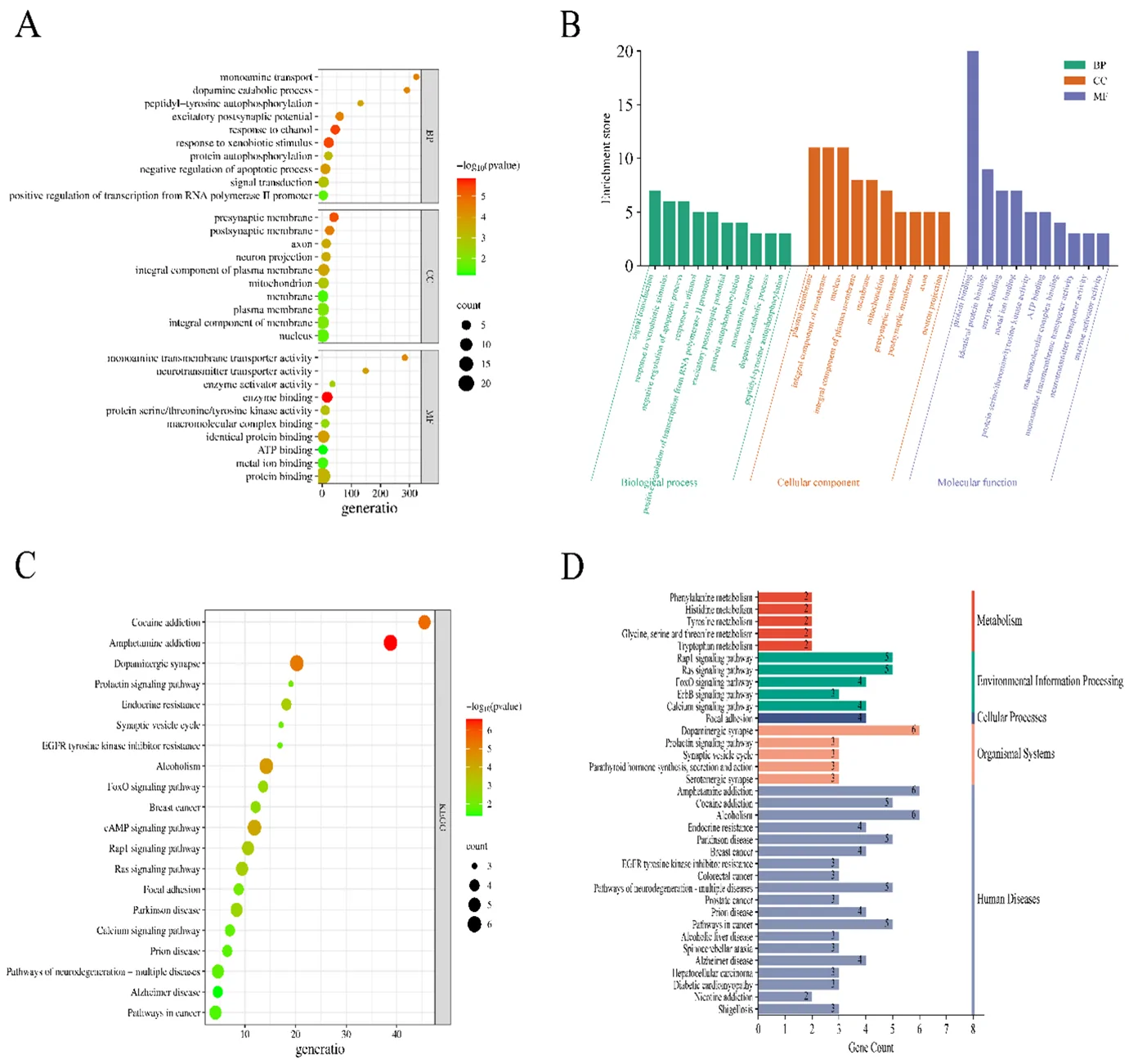
**Gene Ontology (GO) and Kyoto Encyclopedia of Genes and Genomes (KEGG) enrichment analyses of ketamine, depression, and cognitive impairment (CI)**. (A) GO enrichment analysis yielded bubble charts displaying the top 10 biological process (BP), cellular component (CC), and molecular function (MF) category terms. The gene ratios and full names of the BP, CC, and MF are depicted on the X-axis and Y-axis, respectively, while the color and size of each bubble indicate the *p*-value and gene count, respectively. (B) GO enrichment analysis was used to generate a histogram with the highest 10 terms in the BP, CC, and MF categories, denoted by green, orange, and purple bars, respectively. (C) The KEGG enrichment analysis yielded a bubble chart showcasing the top 20 pathways. (D) Identification of the KEGG classification for all the 35 KEGG pathways identified.

We used the GO database to analyze the functional enrichment of 20 common targets. We further investigated the various ways that ketamine was able to elicit cognition improvements and antidepressant effects. We identified 145 items categorized for BP (78), MF (39), and CC (28). Fig. 3A,B show bubble charts of the top-10 enriched BP, CC, and MF terms, including several biological processes, such as response to xenobiotic stimulus, signal transduction, response to ethanol, positive regulation of transcription from RNA polymerase II promoter, negative regulation of apoptotic processes, and excitatory postsynaptic potential. The plasma membrane primarily enriched common targets, including the integral member and nucleus components as well as integral components of the plasma membrane and several regions of the membrane. We observed that molecular functions were enriched primarily in protein and identical protein binding, metal ion and enzyme binding, and protein serine-threonine-tyrosine kinase activity.

### 3.6 KEGG Database Analysis of Signaling Pathways Enrichment of Genes

Analyses of signaling-pathway enrichment were conducted on 20 common targets using *p* < 0.05 (Table 1). A total of 35 KEGG pathways that markedly enriched 20 of the most common targets were identified. Fig. 3C,D illustrate bubble charts of the top-20 signaling pathways, according to gene counts. The signaling pathways with the highest gene counts were as follows: “amphetamine addiction” (hsa05031, n = 6), which involves dopaminergic neurotransmission (a crucial process for reward and mood regulation), dysregulation of which is often linked to depressive symptoms and cognitive deficits [32,33]; “dopaminergic synapse” (hsa04728, n = 6), which regulates dopamine signaling, influencing mood, motivation, and executive function (ketamine may modulate dopaminergic activity, contributing to its antidepressant and cognition-enhancing effects) [34,35]; “alcoholism” (hsa05034, n = 6) and “cyclic adenosine monophosphate (cAMP) signaling pathway” (hsa04024, n = 6), which plays a role in neuronal plasticity and memory formation, both of which are often impaired in depression (ketamine may enhance synaptic connectivity through this mechanism) [36]; and “cocaine addiction” (hsa05030, n = 5), “Rap1 signaling pathway” (hsa04015, n = 5), and “Ras signaling pathway” (hsa04014, n = 5), which are involved in the regulation of cell migration, survival, and synaptic connectivity, playing critical roles in neurodevelopment and stress responses [37].

**Table 1.** **Signaling-pathway enrichment of key genes involved in depression and cognitive impairment**.

ID	Term	Count	Gene	*p*
hsa05031	Amphetamine addiction	6	*GRIN2A, MAOB, MAOA, SIRT1, SLC6A3, GRIN1*	2.45 × 10^−⁢6^
hsa04728	Dopaminergic synapse	6	*GSK-3β, GRIN2A, MAPK8, MAOB, MAOA, SLC6A3*	6.22 × 10^−⁢6^
hsa05034	Alcoholism	6	*GRIN2A, MAOB, ADORA2A, MAOA, SLC6A3, GRIN1*	3.47 × 10^−⁢5^
hsa04024	cAMP signaling pathway	6	*GRIN2A, MAPK8, ADORA2A, PDE4D, PDE4A, GRIN1*	8.19 × 10^−⁢5^
hsa05030	Cocaine addiction	5	*GRIN2A, MAOB, MAOA, SLC6A3, GRIN1*	2.86 × 10^−⁢6^
hsa04015	Rap1 signaling pathway	5	*GRIN2A, ADORA2A, EGFR, GRIN1, IGF1R*	8.57 × 10^−⁢4^
hsa04014	Ras signaling pathway	5	*GRIN2A, MAPK8, EGFR, GRIN1, IGF1R*	0.001
hsa05012	Parkinson’s disease	5	*MAPK8, MAOB, ADORA2A, MAOA, SLC6A3*	0.002
hsa05022	Pathways of neurodegeneration – multiple diseases	5	*GSK-3β, GRIN2A, MAPK8, SLC6A3, GRIN1*	0.016
hsa05200	Pathways in cancer	5	*GSK-3β, MAPK8, EGFR, ESR2, IGF1R*	0.023
hsa01522	Endocrine resistance	4	*MAPK8, EGFR, ESR2, IGF1R*	0.001
hsa04068	FoxO signaling pathway	4	*MAPK8, SIRT1, EGFR, IGF1R*	0.002
hsa05224	Breast cancer	4	*GSK-3β, EGFR, ESR2, IGF1R*	0.003
hsa04510	Focal adhesion	4	*GSK-3β, MAPK8, EGFR, IGF1R*	0.009
hsa04020	Calcium signaling pathway	4	*GRIN2A, ADORA2A, EGFR, GRIN1*	0.015
hsa05020	Prion disease	4	*GSK-3β, GRIN2A, MAPK8, GRIN1*	0.019
hsa05010	Alzheimer’s disease	4	*GSK-3β, GRIN2A, MAPK8, GRIN1*	0.046
hsa04917	Prolactin signaling pathway	3	*GSK-3β, MAPK8, ESR2*	0.009
hsa04721	Synaptic vesicle cycle	3	*SLC6A2, SLC6A3, SLC6A4*	0.012
hsa01521	EGFR tyrosine kinase inhibitor resistance	3	*GSK-3β, EGFR, IGF1R*	0.012

EGFR, epidermal growth factor receptor; cAMP, cyclic adenosine monophosphate.

**Fig. 4.**
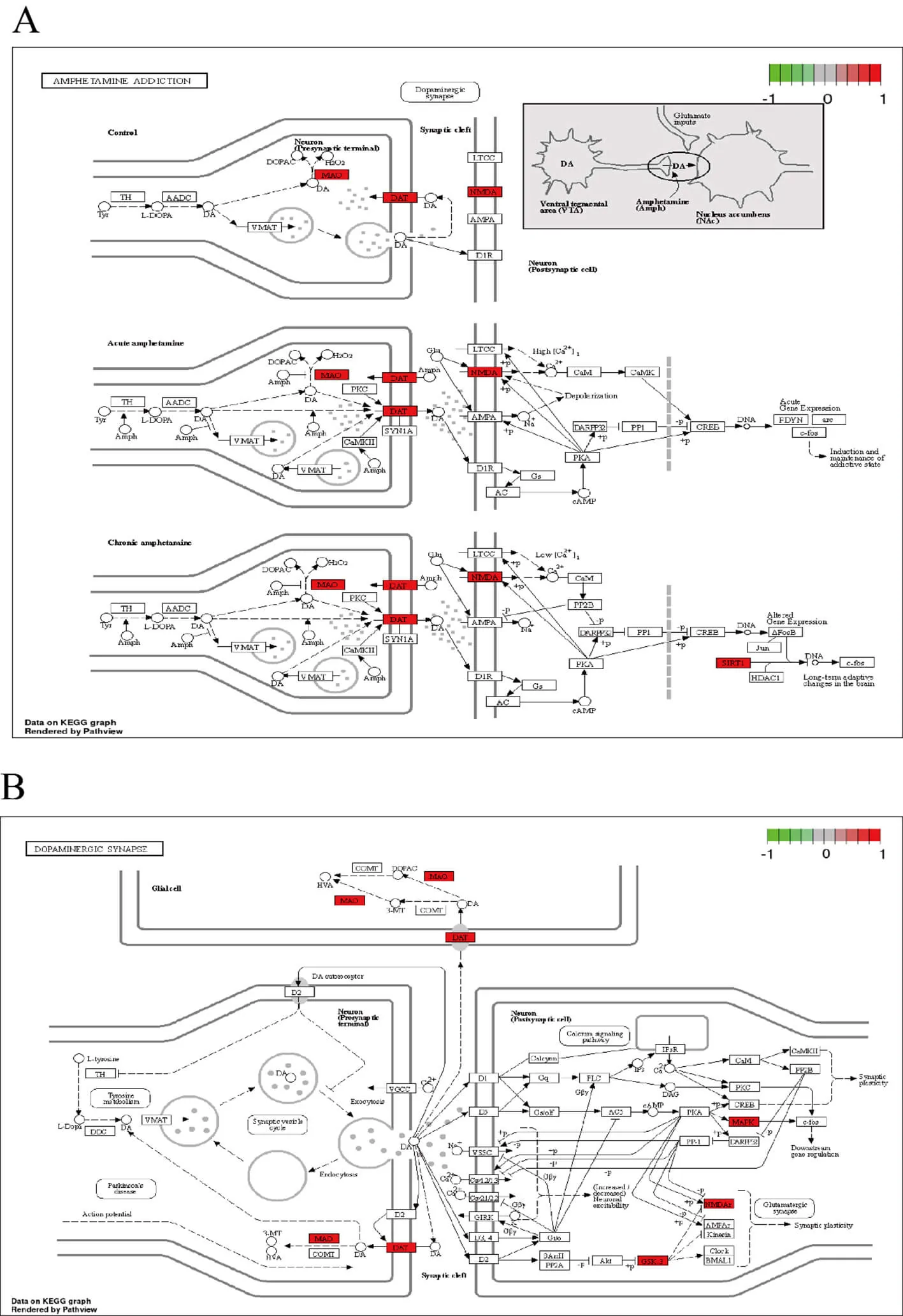
**Distribution of key targets in the two most relevant pathways**. (A) Dispersion of crucial targets within the amphetamine addiction pathway. (B) Dispersion of crucial targets within the dopaminergic synapse pathway.

Specifically, the pathways of “amphetamine addiction” and “dopaminergic synapse”, which encompassed most genes, may serve as crucial pathways for the positive effects of ketamine upon depression and CI. As shown in Fig. 4A,B, we visualized two signaling pathways with Pathview (https://bioconductor.org/packages/devel/bioc/html/pathview.html) [38].

### 3.7 Network Construction and Analyses 

**Fig. 5.**
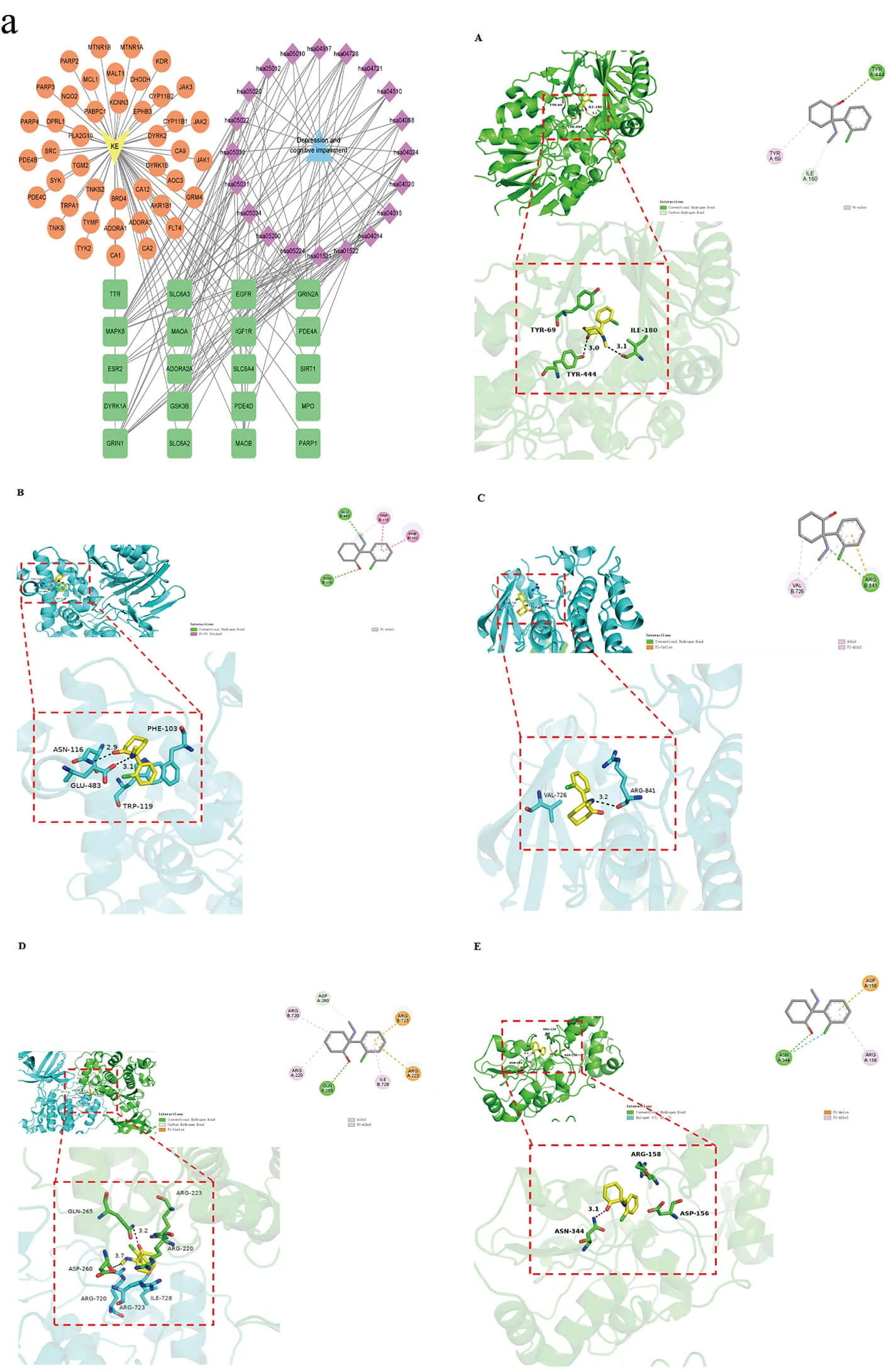
**Ketamine–target–disease-pathway network and molecular docking results of ketamine with core targets**. (a) Ketamine–target–disease-pathway network. The yellow inverted triangle represents ketamine, orange circles denote targets, blue triangle represents disease, purple rhombi denote signaling pathways, and green rectangles represent common genes. Molecular docking process. (A) MAO-A and ketamine; (B) MAO-B and ketamine; (C) EGFR and ketamine; (D) GSK-3β and ketamine; and (E) SIRT1 and ketamine. KE, Ketamine.

We calculated a network with 85 nodes and 173 edges using Cytoscape 3.7.1 software. We constructed a diagram for the two diseases with the drug-target-pathway-disease network diagram, which featured one drug, 20 potential signaling pathways, and 20 shared targets. According to the data, ketamine demonstrated its therapeutic effects through several targets, in which the impact of ketamine on the disease was essential in both dopaminergic synaptic and amphetamine addiction pathways, as shown in Fig. 5a.

### 3.8 Molecular Docking

We used molecular docking to analyze the active targets and drugs to verify our network pharmacology results. We studied the interaction with ketamine and the key target genes, including *MAOA*, *MAOB*, *EGFR*, *GSK-3β*, and *SIRT1*. For the molecular docking, we used AutoDock Vina. To identify the proteins connected to these five genes, we used PDB. The IDs were as follows: *MAOA* (PDB ID: 2BXR), *MAOB* (PDB ID: 1OJA), *EGFR* (PDB ID: 6TG0), *GSK-3β* (PDB ID: 1J1B), and *SIRT1* (PDB ID: 4BN4). Fig. 5A–E show a three-dimensional illustration of the hydrogen bonds (see also Table 2).

**Table 2.** **Molecular docking of the key targets associated with ketamine and depression and cognitive impairment**.

Gene	Target	Docking score (kcal/mol)
*MAOA*	2BXR	–7.2
*MAOB*	1OJA	–7.0
*EGFR*	6TG0	–6.6
*GSK-3β*	1J1B	–7.5
*SIRT1*	4BN4	–7.2

MAO-A/MAO-B, Monoamine oxidase; GSK-3β, Glycogen synthase kinase-3β; SIRT1, Sirtuin-1.

For 2BXR (Fig. 5A), ketamine could form hydrogen bonds with tyrosine (TYR)444 (length = 3.0 Å) and isoleucine (ILE)180 (length = 3.1 Å). For 1OJA (Fig. 5B), ketamine could form hydrogen bonds with asparagine (ASN)116 (length = 2.9 Å) and glutamine (GLU)483 (length = 3.1 Å). For 6TG0 (Fig. 5C), ketamine could form a hydrogen bond with arginine (ARG)841 (length = 3.2 Å). For 1J1B (Fig. 5D), ketamine could form a hydrogen bond with glutamine (GLN)265 (length = 3.2 Å) and asparagine (ASP)260 (length = 3.7 Å). For 4BN4 (Fig. 5E), ketamine could form a hydrogen bond with ASN344 (length = 3.1 Å).

## 4. Discussion

A clinical study conducted in 2000 revealed the enduring antidepressant impact of minimal amounts of ketamine [39]. Two 2018 articles published in *Nature* offer insight into the means behind ketamine’s antidepressant effect [40]. This finding is of importance in the field of clinical psychiatry. In this study, we examined ketamine’s action to treat patients with CI and depression. We used molecular docking and network pharmacology to offer a theoretical foundation to identify ketamine’s new mechanisms of action and pharmacological targets.

We screened 63 potential ketamine targets from the TCMSP, SymMap, HERB, HIT, and SwissTargetPrediction databases. We then acquired 1874 depression targets and 1983 CI targets from the GeneCards, OMIM, and DisGENET databases. Applying bioinformatic methods, 20 potential targets of ketamine for people with depression and CI were systematically obtained. To examine the interactions among proteins and to identify key targets, we created a PPI network. Topological analysis revealed that five central target genes (*MAOA*, *MAOB*, *GSK-3β*, *SIRT1*, *EGFR*) may have a significant impact on the antidepressant effects of ketamine and the ability to improve CI.

GSK-3β is essential in neuronal growth, glucose metabolism, and tissue repair [41]. GSK-3β is a serine–threonine kinase and is part of the phosphatidylinositol-3-kinase/protein kinase B (PI3K/Akt1) signaling pathway. It is controlled by brain-derived neurotrophic factor (BDNF), glutamate receptors, and neuregulin-1 [42]. GSK-3β is essential in the pathophysiology of mood disorders [43]. Its activation is associated with mania-like and depressive behaviors in animals. GSK-3β regulates neuronal survival, axonal growth, and synaptic plasticity via the PI3K/Akt1 signaling pathway, processes closely associated with learning and memory. Studies have demonstrated that hyperactivation of GSK-3β impairs synaptic structure and function, contributing to cognitive deficits [44,45,46,47]. Activation of GSK-3β induces neuroinflammation and oxidative stress, which are critical contributors to both depression and cognitive disorders. By inhibiting GSK-3β, ketamine may reduce the release of inflammatory mediators, thereby mitigating cognitive dysfunction [48]. According to our results, GSK-3β is essential in improving CI and regulating depression.

SIRT1, which is a nicotinamide adenine dinucleotide-dependent histone deacetylase [49]. Studies have associated SIRT1 expression with MDD. Research on MDD involving SIRT1 has focused on the hippocampus, medial prefrontal cortex, and nucleus accumbens. These regions of the brain are associated with cognitive symptoms and emotional control. Libert and Lei found that mice without SIRT1 demonstrated cognitive decline and depressive behavior [50,51]. Abe-Higuchi et al. [52] showed that expression of SIRT1 was reduced by chronic stress in the dentate gyrus of the mouse hippocampus, which contributed to behaviors that were similar to depression.

MAO is a naturally occurring enzyme in the human body. It plays a crucial part in decomposing various monoaminergic neurotransmitters in the brain [53]. MAO has two isoforms, each produced by separate genes. MAO-A is found in catecholaminergic neurons, whereas MAO-B is found in histaminergic neurons, serotonergic neurons, and glial cells [54]. Both isoforms can deactivate monoamine neurotransmitters. MAOIs increase norepinephrine, dopamine, and serotonin levels in the body and thus play an important role in mood regulation, cognitive enhancement, and monoamine effects [55].

According to studies of depression in humans, monoamine oxidase inhibitors (MAOI) levels in rodent models in the anterior and prefrontal cingulate cortex are related to suicidal ideation, depression, and neurocognitive impairment. Those findings indicate a potential intrinsic connection between depressive symptoms and abnormalities in the metabolism of monoamine neurotransmitters. Moreover, several studies have provided evidence of synaptic dysfunction and structural changes in relation to depression [56]. Histopathologic and postmortem morphometric studies have shown a smaller pyramidal neuron size and a limited number of hippocampal granule cells in the orbitofrontal and dorsolateral prefrontal cortex. As a result, cortical thickness in people with depression had a corresponding reduction [57]. On the basis of immunohistochemistry, electron microscopy, and RNA sequencing, the number of synapses decreased, as did functional connections. Related genes and proteins in the hippocampus, prefrontal cortex, and nucleus accumbens were also limited in people experiencing depression [58]. The presence of these lesions worsened depressive symptoms and cognitive disorders to different extents.

A growth factor is a type of cytokine that plays an important role in regulating cell development, migration, and survival in brain tissue [59]. Modifications in the blood concentrations of BDNF, epidermal growth factor (EGF), erythropoietin, fibroblast growth factor, insulin-like growth factor, nerve growth factor, transforming growth factor-β, and vascular endothelial growth factor have been found to be associated with the severity of psychiatric symptoms, the risk of recurrence, impairment in social skills, and decline in cognitive abilities [60]. A study in individuals with MDD demonstrated that cognitive changes in patients with MDD are linked to a specific genetic variation (rs2250724) in *EGF* [61]*.* Another study showed that patients with MDD had increased expression of Erb-b2 receptor tyrosine kinase-3 and significant improvement in depressive symptoms after 12 weeks of antidepressant medication [62]. Sohan and colleagues found that people with MDD generally had a lower level of EGF in the blood [63]. Notably, women suffered a greater reduction in the EGF level as the severity of depression intensified [63]. EGF activates protein tyrosine kinases and triggers diverse intracellular signaling pathways. Based on the neurotrophic-factor hypothesis, EGF signaling plays a regulatory role in the development of dopaminergic neurons and monoamine metabolism [64]. EGFR is an essential target for the antidepressant impact of ketamine and its ability to enhance CI, according to our hypothesis.

We analyzed signaling and functional pathway enrichment of genes using KEGG and GO databases, respectively. According to the functional enrichment analysis, we identified significant biological processes related to ketamine to treat CI and depression. These processes included transmembrane transport of substances, cellular signal transduction, regulation of enzyme activity, apoptosis, inflammation, and oxidative stress.

The dopaminergic synapse and amphetamine-addiction pathways have an essential antidepressant impact on ketamine and on CI improvement. Amphetamines have a long-lasting impact on dopaminergic, noradrenergic, serotonergic, and glutamatergic systems [65]. The brains of patients addicted to amphetamine display impaired dopamine terminal function and activation of specific neuroinflammatory pathways [66]. These pathways involve cytokines and chemokines released by microglia, which lead to amphetamine-induced neuronal damage and neuropsychiatric disorders, as well as cognitive deficits (including impaired executive and memory function), depression, and anxiety [67]. Patients experiencing depression and CI have been found to have a higher risk of developing Parkinson’s disease [68]. According to rodent research, low-dose ketamine in the prefrontal cortex improves the circulation and release of glutamate [69]. Patients experiencing depression have reduced glutamatergic and GABAergic receptor expression in the prefrontal cortex as well as reduced dendritic spine density. As a result, ketamine treatment stimulates α-amino-3-hydroxy-5-methyl-4-isoxazole propionic acid receptors (AMPAR) by facilitating glutamate release in the prefrontal cortex. This stimulation subsequently triggers the downstream mammalian target of rapamycin signaling pathway, which promotes increased expression of BDNF and synapse formation and ultimately enhances glutamate transmission and produces an antidepressant effect. Increased AMPAR activation and glutamate release in the prefrontal cortex are essential in relation to the impact of ketamine on depression. Our results verified our hypothesis that the dopaminergic synapse and amphetamine-addiction pathways are essential to the impact of ketamine on depression and CI improvement. Our results suggest that ketamine could be used to treat depression and CI, but additional *in vitro* experiments are needed to validate our data.

According to our results, neurotransmitter receptors that are abnormal or signaling pathways that have defects in the thalamus and cerebral cortex may have a negative effect on the brain’s ability to respond to mental stimulation. This impairment may result in CI and depression. Currently, depression is treated primarily with drugs that target monoamine molecules, such as serotonin, norepinephrine, and dopamine. Selective serotonin reuptake inhibitors are efficacious in treating depression, but often they are poorly tolerated and have significant side-effects. Antidepressant medications are associated with several challenges, including extended treatment duration, delayed therapeutic effects, significant adverse reactions, and limited efficacy in a subset of individuals [70]. These limitations suggest that these drugs might only exert an indirect influence and fail to precisely target the underlying mechanisms of depression. Among antipsychotic agents, ketamine stands out as an efficacious and safe drug. Investigation into the antidepressant effects and CI of ketamine is a promising research direction in pharmacology.

Based on the data from network pharmacology, molecular docking was undertaken to investigate the combination of five hub genes (*MAOA*, *MAOB*, *GSK-3β*, *SIRT1*, *EGFR*) and ketamine. Molecular docking revealed a range in binding affinity from –6.6 to –7.9 kcal/mol, suggesting that all targets possessed favorable binding ability with ketamine. Among the five target proteins, MAO-A, MAO-B, SIRT1, and EGFR exhibited relatively lower binding affinities, with EGFR exhibiting the lowest binding energy, indicating a highly stable binding interaction between ketamine and EGFR.

The main limitations of this study are as follows:

1. Ketamine’s therapeutic effects on cognition and depression are mediated by various pathways and targets. We did not, however, investigate other pathways and targets.

2. We used data-mining methods. To verify these findings, however, animal experiments and clinical trial would be required.

3. Ketamine has been approved clinically and has a safety profile that is well established. Its safety and efficacy in treating patients who have depression, however, have not been evaluated thoroughly.

## 5. Conclusions

We have preliminarily identified key proteins associated with ketamine, including MAO-A, MAO-B, GSK-3β, SIRT1, and EGFR, as well as critical signaling pathways, including amphetamine addiction, dopaminergic synapse, and the cAMP signaling pathway, in alleviating depression and CI. The results of this study demonstrated how ketamine can improve symptoms of CI and depression.

## Data Availability

The data sets related to this study are available from the corresponding author on reasonable request.
